# Evaluation of viscosities of typical drainage fluids to promote more evidence-based catheter size selection

**DOI:** 10.1038/s41598-023-49160-8

**Published:** 2023-12-13

**Authors:** Erkan Celik, Lukas Goertz, Joern Henze, Markus Schütz, Ben Mink, Sebastian Brinkmann, Hans-Ulrich Laasch, Annette M. Schmidt, Holger Grüll, David Maintz, Roman Kloeckner, Florian Lorenz, Daniel Pinto dos Santos, Seung-Hun Chon

**Affiliations:** 1 Department of Diagnostic and Interventional Radiology, Faculty of Medicine and University Hospital Cologne, University of Cologne, Kerpener Str. 62, 50937 Cologne, Germany; 2 Department of Chemistry, Faculty of Mathematics and Natural Sciences, Institute for Physical Chemistry, University of Cologne, Greinstraße 4-6, 50939 Cologne, Germany; 3 Department of General, Visceral, Cancer and Transplant Surgery, Faculty of Medicine and University Hospital Cologne, University of Cologne, Kerpener Str. 62, 50937 Cologne, Germany; 4 Department of General and Visceral Surgery, St. Josef-Hospital Bochum, Ruhr-University Bochum, Gudrunstrasse 56, 44791 Bochum, Germany; 5 Minnova Medical Foundation CIC, Wilmslow, UK; 6 Institute of Interventional Radiology, University Hospital Schleswig-Holstein, Campus Lübeck, Ratzeburger Allee 160, 23562 Lübeck, Germany; 7 Interdisciplinary Endoscopy Unit, Department of Gastroenterology and Hepatology, Faculty of Medicine and University Hospital Cologne, University of Cologne, Kerpener Str. 62, 50937 Cologne, Germany; 8 Department of Radiology, University Hospital Frankfurt, Theodor-Stern-Kai 7, 60590 Frankfurt, Germany

**Keywords:** Bacterial infection, Biliary tract disease, Pancreatitis

## Abstract

Percutaneous drainage is a first-line therapy for abscesses and other fluid collections. However, experimental data on the viscosity of body fluids are scarce. This study analyses the apparent viscosity of serous, purulent and biliary fluids to provide reference data for the evaluation of drainage catheters. Serous, purulent and biliary fluid samples were collected during routine drainage procedures. In a first setup, the apparent kinematic viscosity of 50 fluid samples was measured using an Ubbelohde viscometer. In a second setup, the apparent dynamic viscosity of 20 fluid samples obtained during CT-guided percutaneous drainage was measured using an in-house designed capillary extrusion experiment. The median apparent kinematic viscosity was 0.96 mm^2^/s (IQR 0.90–1.15 mm^2^/s) for serous samples, 0.98 mm^2^/s (IQR 0.97–0.99 mm^2^/s) for purulent samples and 2.77 mm^2^/s (IQR 1.75–3.70 mm^2^/s) for biliary samples. The median apparent dynamic viscosity was 1.63 mPa*s (IQR 1.27–2.09 mPa*s) for serous samples, 2.45 mPa*s (IQR 1.69–3.22 mPa*s) for purulent samples and 3.50 mPa*s (IQR 2.81–3.90 mPa*s) for biliary samples (all differences *p* < 0.01). Relative to water, dynamic viscosities were increased by a factor of 1.36 for serous fluids, 2.26 for purulent fluids, and 4.03 for biliary fluids. Serous fluids have apparent viscosities similar to water, but biliary and purulent fluids are more viscous. These data can be used as a reference when selecting the drainage catheter size, with 8F catheters being appropriate for most percutaneous drainage cases.

## Introduction

Image-guided percutaneous drainage is a well-established, minimally invasive treatment alternative to surgical removal of pathological fluid collections^1–3^. The effectiveness of this method is well documented in the literature^4–8^. Among the various imaging modalities, computed tomography (CT) is preferred by many clinicians for interventional procedures due to its high temporal and spatial resolution. In particular, CT is the modality of choice for deep-seated locations or when fluid collections are surrounded by sensitive structures^9^. In the absence of guidelines on which catheter size to use, the choice is not evidence-based but based on personal preference and experience. Many physicians prefer larger catheters, but this can increase patient discomfort, procedure time and the risk of complications, such as the need for more dilation of the access tract and an increased bleeding risk^10^. There is a paucity of scientific data on the relationship between drain size and drainage efficiency, and the suitability of the current drain sizing system has been questioned^11,12^. Among other factors influencing the technical success of percutaneous catheter drainage, the viscosity of the fluid and the amount and size of solid particles in the fluid should be considered when discussing the required catheter size. The aim of this study was to analyze the apparent viscosities of the drainage fluid samples obtained and to compare the specific properties with model fluids such as water and glycerol. These measurements can be used as a basis for the selection of drainage catheter sizes as well as for the development and evaluation of new drainage catheters.

## Results

### Experimental setup 1

Of the 50 fluid samples, 31 were classified as serous, 15 as purulent and 4 as biliary (Table 1).

**Table 1:** Kinematic and dynamic apparent viscosity of serous, purulent and biliary samples. Experimental setup 1 was performed at 37 °C, while experimental setup was performed at room temperature (22.5 °C).

Fluid type	Experimental setup 1 (apparent kinematic viscosity)		Experimental setup 2 (apparent dynamic viscosity)	
	n [failed]	Median/IQR [mm^2^/s]	n [failed]	Median/IQR [mPa*s]
Serous	31 [0]	0.96 [0.90–1.15]	4 [0]	1.63 [1.27–2.09]
Purulent	15 [13]	0.98 [0.97–0.99]	11 [1]	2.45 [1.69–3.22]
Biliary	4 [0]	2.77 [1.75–3.70]	5 [1]	3.50 [2.81–3.90]

*IQR* interquartile range, mPa*s millipascal-second.

Of the 15 purulent samples, the capillary of the Ubbelohde viscometer was blocked by debris or solid components in 13 cases and the measurement failed. In the remaining cases, the median apparent kinematic viscosity measured by the Ubbelohde viscometer was 0.96 mm^2^/s (IQR 0.90–1.15 mm^2^/s) for serous samples, 0.98 mm^2^/s (IQR 0.97–0.99 mm^2^/s) for purulent samples and 2.77 mm^2^/s (IQR 1.75–3.70 mm^2^/s) for biliary samples.

### Experimental setup 2

Of the 20 cases included in this study, 11 were classified as purulent, 4 as serous and 5 as biliary (Table 1). The volume of samples collected ranged from 10 to 100 ml. Two samples were excluded from further analysis for technical reasons (in both cases there was not enough material to perform a second measurement). The median apparent dynamic viscosity was 1.63 mPa*s (IQR 1.272.09 mPa*s) for serous samples, 2.45 mPa*s (IQR 1.69–3.22 mPa*s) for purulent samples and 3.50 mPa*s (IQR 2.81–3.90 mPa*s) for biliary samples.

### Comparison to model fluids across both setups

To make the results more comparable, we compared the apparent kinematic and dynamic viscosities measured in Experiments 1 and 2 with those of water and glycerol, which were measured as controls in the two experimental setups. The measured kinematic viscosity was 0.70 mm^2^/s for water and 401 mm^2^/s for glycerol at 37 °C. The measured dynamic viscosity was 0.94 mPa*s for water and 925 mPa*s for glycerol at 22.5 °C. Table 2 shows the relative viscosities of the liquid samples with respect to water and glycerol. Relative to water, the apparent kinematic viscosities were increased by a factor of 1.38 (IQR 1.29–1.64) for serous fluids, 1.41 (IQR 1.38–1.43) for purulent fluids, and 3.98 (IQR 2.51–5.31) for biliary fluids. Apparent dynamic viscosities were increased by a factor of 1.36 (IQR 1.34–1.99) for serous fluids, 2.26 (IQR 1.70–3.39) for purulent fluids, and 4.03 (IQR 3.73–4.29) for biliary fluids compared to water.

**Table 2:** Comparison of apparent kinematic (experimental setup 1, 37 °C) and dynamic (experimental setup 2, 22.5 °C) viscosities of serous, purulent and biliary fluid samples water and glycerol.

Fluid type	Relative kinematic viscosity, median [IQR]	Relative dynamic viscosity, median [IQR]
Relative to water		
Serous	1.38 [1.29–1.64]	1.36 [1.34–1.99]
Purulent	1.41 [1.38–1.43]	2.26 [1.70–3.39]
Biliary	3.98 [2.51–5.31]	4.03 [3.73–4.29]
Relative to glycerol (10^–3^)		
Serous	3.42 [3.21–4.09]	1.47 [1.45–2.15]
Purulent	3.50 [3.45–3.55]	2.44 [1.84–3.66]
Biliary	9.91 [6.26–13.23]	4.35 [4.03–4.63]

The measured kinematic viscosity was 0.70 mm^2^/s for water and 401 mm^2^/s for glycerol at 37 °C. The measured dynamic viscosity was 0.94 mPa*s for water and 925 mPa*s for glycerol at 22.5 °C.

*IQR* interquartile range.

**Figure 1:**
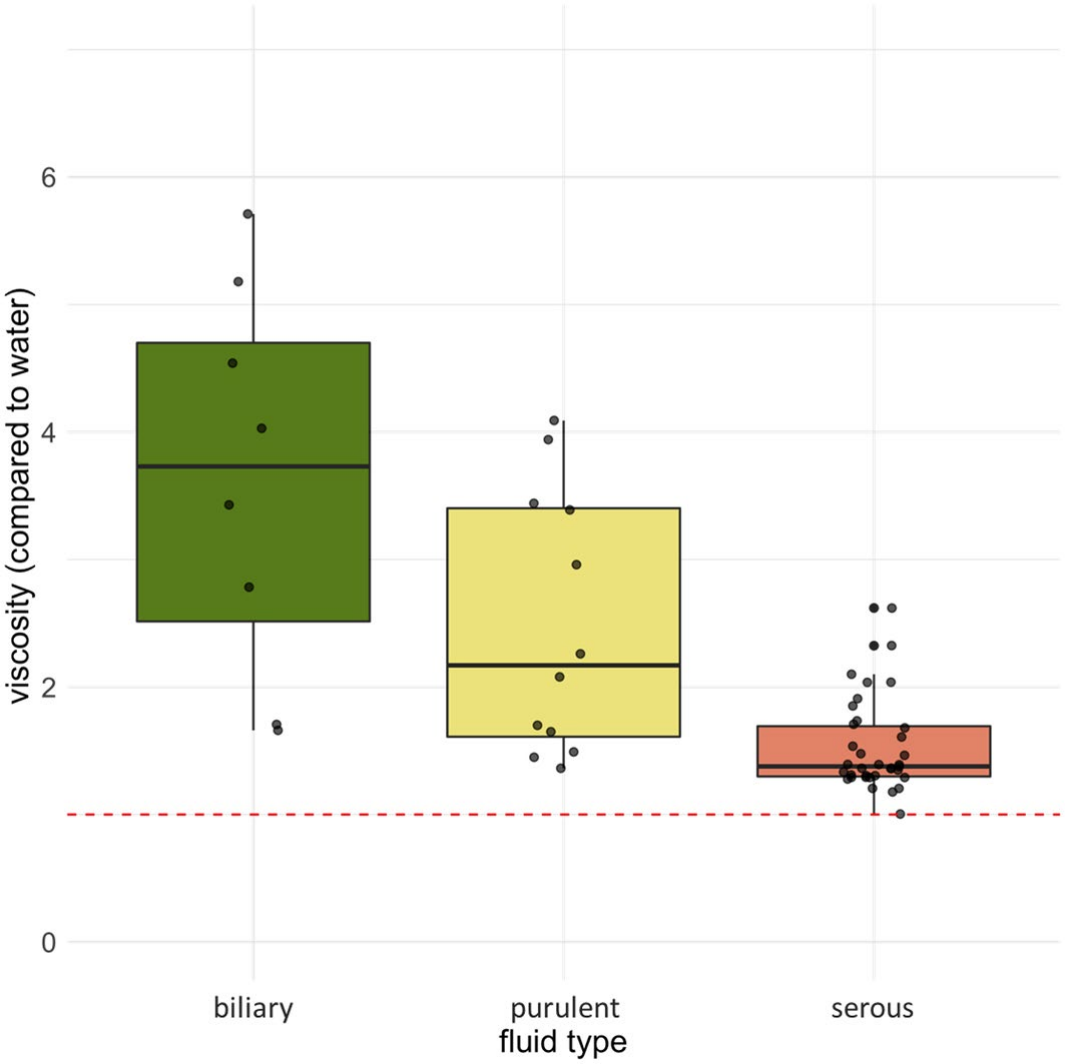
Boxplots for relative viscosity of biliary, purulent and serous samples in comparison to water.

ANOVA with Tukey post-hoc test showed statistically significant differences between the relative viscosities to water of all three fluid types (purulent vs. biliary: *p* = 0.006, serous vs. biliary: *p* < 0.001, serous vs. purulent: *p* = 0.002), as visualized in Fig. 1.

**Figure 2:**
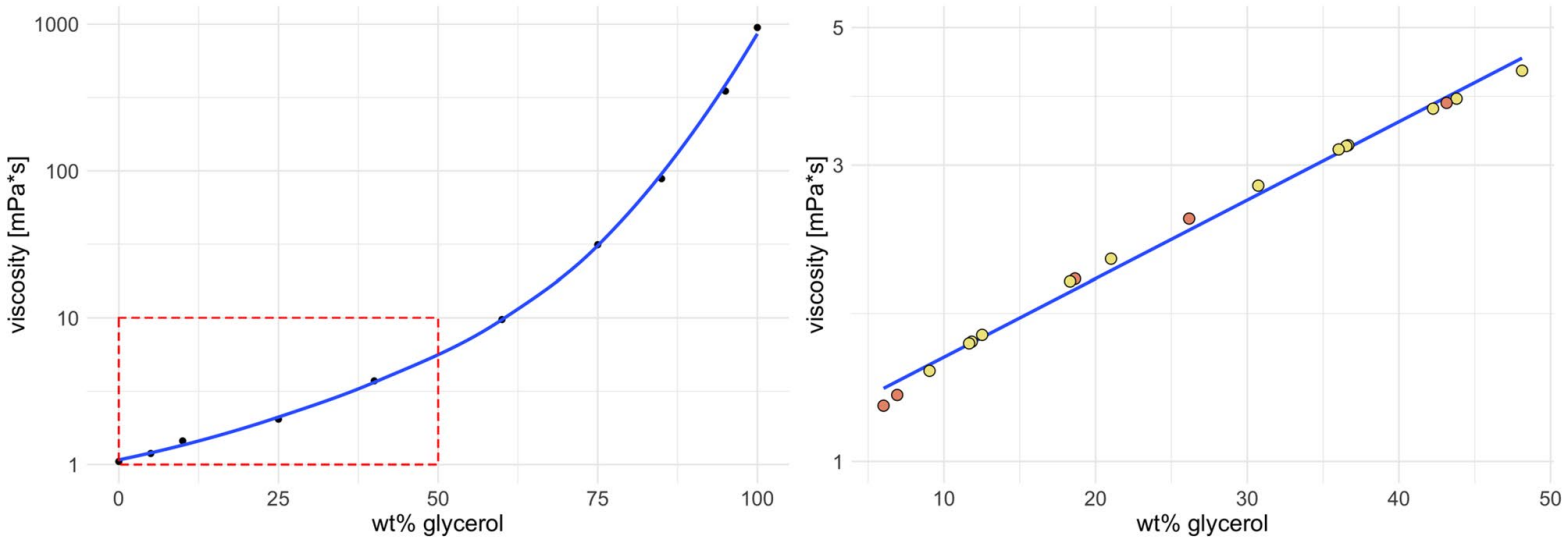
Comparison of the viscosity of water/glycerol mixtures as a function of the weight percentage (wt%) of glycerol and the viscosity of the measured samples in comparison. The window is zoomed to a maximum of 50% w/w glycerol, as no sample exceeded the viscosity of this mixture, further indicating the suitability of glycerol as a positive control.

By way of comparison, Fig. 2 shows the viscosity of water/glycerol mixtures as a function of the weight percentage (wt%) of glycerol and the viscosity of measured samples in comparison.

## Discussion

The experiments in this manuscript were designed to determine the apparent viscosities of biological fluids that are frequently drained in clinical routine. As biological fluids can contain multiple fluid phases and even debris, the results should be considered indicative of the apparent Newtonian viscosities of these different fluids. Taking these considerations into account, the results showed that the median apparent viscosities of purulent and biliary samples were 1.4–2.3 times and 4.0 times the viscosity of water, respectively, while the apparent viscosity of serous fluids was only slightly higher (factor 1.3). This finding may provide a basis for a more evidence-based discussion about the appropriate size of catheter tube for percutaneous drainage. There are a number of arguments for using smaller or larger drainage tube sizes. Some clinicians prefer larger catheter diameters (> 9 French [Fr]), arguing that a larger diameter results in higher flow and therefore better drainage efficacy, particularly for more viscous fluids^13^. There is also evidence that larger tubes (12–14 Fr) may be more stable and less prone to kinking and blockage, whereas the stability of catheters depends mainly on the material of the catheter (usually kink-resistant polyurethane)^14^. Arguments for smaller drainage tube sizes (7–8 Fr) include improved patient comfort, lower periprocedural complication rates and shorter procedure times ^10,15^. Patient comfort is particularly important when the drain is in place for prolonged periods and when the drain is placed dorsally, with the patient lying on the catheter most of the time (e.g. retroperitoneal abscesses and nephrostomies), or in the liver, which is constantly moving with respiration. In our experiments, most apparent viscosities were only slightly higher than the viscosity of water, suggesting that aspiration should be possible through smaller tubes such as 8 Fr. These results suggest that a standard 8 Fr drain may be sufficient for most body fluid collections and that the technical efficacy of drainage does not necessarily correlate with the internal diameter of the drain. Actively aspirating the fluid, initially and repeatedly throughout the course of the disease, may be of paramount importance to the success of the efficacy of drainage therapy. The measured high apparent viscosity of bile was of particular interest as 6F and 7F catheters have been the standard for biliary drainage in the UK for many years.

A study by Gobien et al. supports the hypothesis that drain size has no relevant effect on drain efficiency. Using percutaneous drains of different materials and inner diameters ranging from 5 to 18 Fr, the authors found no significant difference in the success rates of intra-abdominal abscess drainage for the different drain types^12^. Park et al. compared the time taken to drain 10 ml of different fluids (water, blood, pseudocyst fluid, abscess fluid, abscess fluid with urokinase) through different catheter sizes (3–13 Fr). They argue that, according to Poiseuille's law, larger catheters provide faster drainage. Indeed, the time to drain 10 ml of abscess fluid could be reduced from > 800 s with a 4 Fr catheter to 100 s with 7 Fr, 50 s with 9 Fr and < 25 s with 11 Fr and 13 Fr. However, even if the flow rate is higher with larger diameters, there is no evidence that this translates into higher drainage efficacy or a better clinical outcome, as high-flow situations in cavity fluids are rare^13^. In a clinical context, we do not believe that drainage efficacy is dependent on higher flow rates. In most cases where drains are placed, the fluid collections are either of a certain size (e.g. abscesses) containing a certain amount of fluid—in which case the time to complete drainage is secondary, as long as it is in the range of a few hours—or placed in low-flow environments (e.g. bile)—in which case even small drains have a higher flow than the fluid produced (often few milliliters per hour). In addition, there are certain clinical conditions (e.g. pleural effusions) where rapid drainage is contraindicated and the use of larger catheters is unnecessary. This is even more important in pneumothorax, as air is approximately 50 times less viscous than water.

This study has several limitations. Firstly, some selection bias cannot be excluded, as all samples could have been aspirated through the clinician-placed percutaneous drainage and some more viscous fluids may not have been included. However, the fact that even undiluted glycerol could be aspirated through an 8 Fr drainage catheter (a single experiment performed outside the present study itself) suggests that this potential selection bias does not limit our findings too much. Second, not only fluid viscosity but also the composition of the fluid itself may play a role in drainage efficacy. In this context, the collected fluids may contain several fluid phases and even small debris, therefore we likely measured the apparent viscosities of the collected fluids, which can differ from the viscosity of single-phase fluids. Moreover, single- and multiple-phase fluids can have different rheometric when assuming diverging baseline parameters. Particularly in cases following pancreatitis, fluid collections may contain solid particles or variable amounts of semi-solid necrotic debris, which carry the risk of clogging the catheter. Our study did not specifically look at particle size. A study by Mithöfer et al. showed that primary percutaneous drainage of pancreatic abscesses was successful in only 31% (9/39), but their data suggest that percutaneous drainage can play an important role in the early management of septic and unstable patients by providing early decompression and evacuation of pus^16^. This is consistent with our own clinical experience. In addition, we did not measure the density of the fluid samples, which would have served as an internal control, since the relationship between kinematic and dynamic viscosity depends on density. Finally, we did not perform an experimental setup to directly compare drainage tube sizes with our model fluids; this should be investigated in an additional series of experiments in the future.

## Conclusions

In conclusion, our study provides insight into the apparent viscosities of commonly used drainage fluids and demonstrates that the apparent viscosities of different body fluids are more or less comparable to water. Therefore, larger drainage tube sizes do not necessarily result in better drainage efficacy, but further series of experiments may be needed to clarify the efficacy of different tube sizes.

## Methods

The study protocol was approved by the local ethics committee of the University Hospital of Cologne (IRB 18-243). Informed consent was obtained from all subjects or their legal guardians for inclusion in this prospective study. The study was conducted in accordance with the STROBE guidelines in compliance with the national legislation and the Code of Ethical Principles for Medical Research Involving Human Subjects of the World Medical Association (Declaration of Helsinki).

### Patient population and specimen collection

All patients included in this study underwent surgical or CT-guided fluid drainage. All fluid samples were collected during standard clinical procedures performed for valid medical indications. No patient underwent additional procedures to be included in this study. For safety reasons, patients with chronic infections (e.g. HIV and hepatitis) were excluded. The following fluid characteristics were recorded: location, volume, quality (purulent, serous or biliary) and drainage tube size. Fluid quality was assessed by consensus between the operator performing the drainage and a second consultant (DP and SH). All drainages were technically successful and no patient was referred for re-drainage.

### CT-guided percutaneous drainage

CT scans were performed before the intervention using a standardized scanning protocol on a multidetector computed tomography scanner (iCT 256 Philips Healthcare, Best, The Netherlands; scanner settings: 120 kV, 400 mAs, slice thickness 0.75 mm). If a fluid collection was suspected, the drainage tube was immediately placed by an interventional radiologist. In this study, 8 Fr, 10 Fr and 12 Fr side-hole pigtail catheters (PerkuCess SGT 8–12 Fr × 30 cm; Peter Pflugbeil GmbH, Zorneding, Germany) were used at the discretion of the interventionalist. The catheter was inserted using the Seldinger technique with an 18 gauge (G) needle for the initial puncture (PerkuCess Ini 18 G × 15 cm; Peter Pflugbeil GmbH, Zorneding, Germany) and a 75 cm, 0.035" guide wire (Amplatz Super Stiff; Boston Scientific Corporation, Boston, MA, USA). Flushing of the drainage tubes with saline three times a day was recommended for all tube sizes.

### Experimental Setups

**Figure 3:**
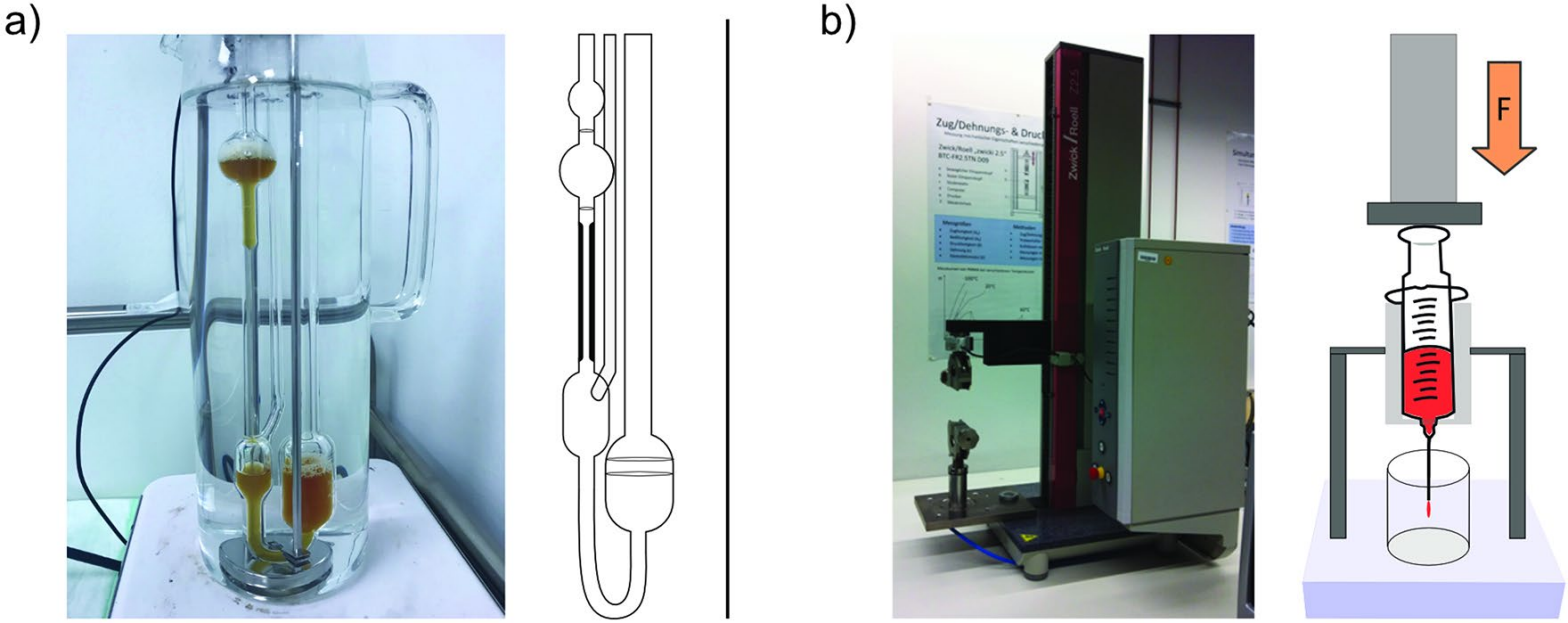
Photographs and diagrams of the test setups: (**a**) the Ubbelohde viscometer and (**b**) in-house designed capillary viscosimeter.

Two experimental setups (Fig. 3) were used to evaluate the apparent viscosities of the collected fluid samples. For both setups, Newtonian behavior of the fluid samples was assumed.

#### Experimental setup 1—apparent kinematic viscosity

Initially, fluid samples from 50 patients obtained between November 2019 and April 2020 either by CT-guided percutaneous drainage (n = 12) or from intraoperative drainages during abdominal surgery (n = 38) were analyzed. Only samples with at least 20 ml of aspirated fluid were included. The apparent kinematic viscosity of the fluids was measured immediately after collection using a calibrated Ubbelohde viscometer (K = 0.01 mm^2^/s^2^; Cannon Instruments, PA, United States) (Fig. 3a). Shear-rates were not evaluated. All measurements were performed under standardized conditions in a water bath heated to 37 °C.

#### Experimental setup 2—apparent dynamic viscosity:

As some measurements of purulent fluids failed in the first experimental setup because the viscometer capillary was too small to allow even small debris to pass under gravity alone, a second setup based on capillary extrusion was employed. Therefore, capillary extrusion experiments are carried out using an in-house designed setup based on the frame of a Zwicki 2.5kN universal testing machine (ZwickRoell GmbH & Co. KG, Ulm, Germany) equipped with a specially designed plunger-capillary system (Fig. 3b). To determine the viscosity of a fluid, it is transferred into a sterile standard 10 ml luer-lock syringe with an internal radius of 7.913 mm connected to a cannula (23 G) of known length (80 mm) and radius (0.195 mm). A uniaxial force is applied to the plunger at a constant velocity using the precision traverse motion of the pulling device at a predetermined speed, causing a constant flow through the capillary/cannula. The precision traverse allows positioning and velocity to be controlled to < 0.03 mm. At the same time, the force is measured using a load cell with an accuracy of < 0.01 N.

The ram speed is varied at a predetermined rate (40, 50 and 60 mm/min). Under the given conditions, this corresponds to a shear rate between 2 × 10^4^ s^−1^ and 3 × 10^4^ s^−1^.

For Newtonian fluids, the Hagen–Poiseuille law can be applied to obtain the fluid velocity from the variation in force measured at a given constant plunger speed.

1 $$\eta = \frac{{{\text{d}}F}}{{{\text{d}}v}} \cdot \frac{{8 \cdot \pi \cdot L_{C} \cdot R_{S}^{4} }}{{R_{C}^{4} }}$$

With $\eta$ = viscosity, u = force, $v$ = velocity, ${L}_{C}$ = length of cannula, ${R}_{S}$ = inner radius of syringe, ${R}_{C}$ = inner radius of cannula and $\frac{\mathrm{d}F}{\mathrm{d}v}$ = slope.

**Figure 4:**
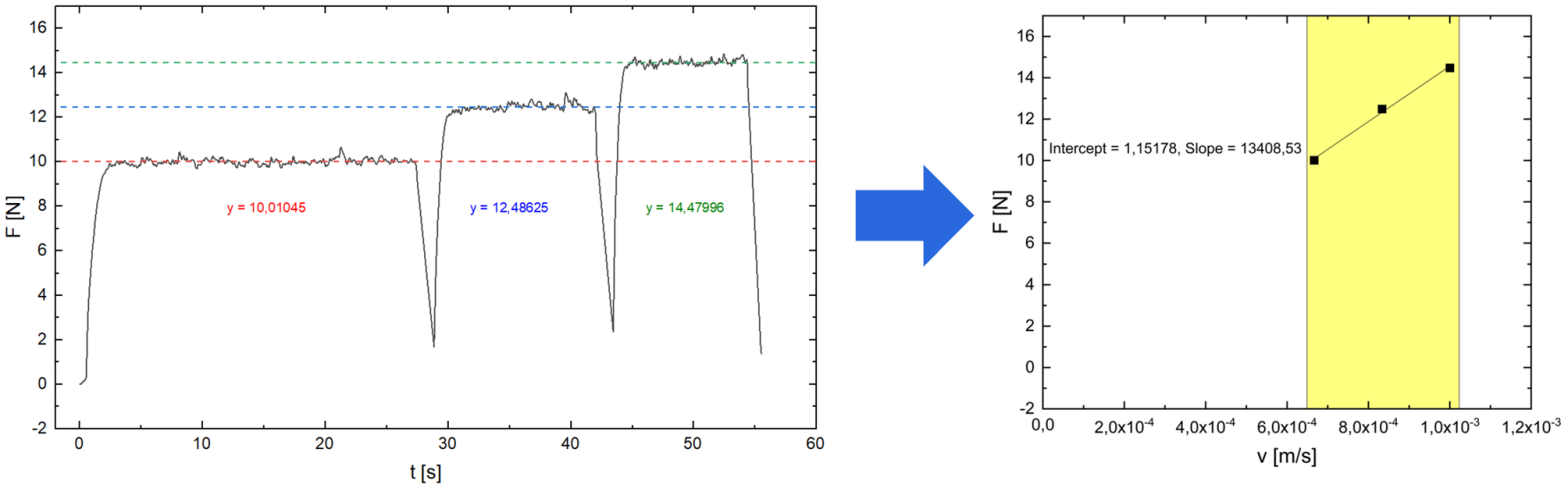
For Experiment 2 (capillary extrusion rheometry), the force required to drive the plunger at a predetermined velocity v is measured using a load cell, and plotted against the plunger velocity v. From the slope dF/dv, the apparent viscosity can be calculated using Eq. 1.

For the biological fluids, we are aware that deviations from Newtonian behavior can occur, but we estimate the viscosity of the fluids under consideration to be approximately constant in the shear rate regime applied and are aware that we are determining an apparent viscosity. The force developed under constant shear is then determined and plotted against piston speed, and the (apparent) viscosity is calculated from the slope (see Fig. 2). This approach has been shown to be of acceptable accuracy in the viscosity range studied by verification on water/glycerol mixtures of various compositions and known viscosity at 22.5 °C. (Fig. 4).

For the second experimental setup, additional fluid samples were collected from 20 patients who underwent CT-guided percutaneous drainage between November 2019 and April 2020. After collection using a 50 ml luer-lock syringe, the samples were stored at room temperature until measurements could be made.

### Statistical analysis

Continuous variables are presented as mean with standard deviation or median and range. Categorical variables are presented as numbers and percentages. Differences between fluid quality groups were assessed by analysis of variance with post-hoc Tukey HSD test. Statistical analysis was performed using R studio version 1.2.5033 (R Core Team 2013). A *p*-value of < 0.05 was considered statistically significant.

## Data Availability

All data will be made available upon request in an anonymized manner (contact: Dr. Daniel Pinto dos Santos, Daniel.pinto-dos-santos@uk-koeln.de).
